# Avoiding Dangerous Missense: Thermophiles Display Especially Low Mutation Rates

**DOI:** 10.1371/journal.pgen.1000520

**Published:** 2009-06-19

**Authors:** John W. Drake

**Affiliations:** Laboratory of Molecular Genetics, National Institute of Environmental Health Sciences, Research Triangle Park, North Carolina, United States of America; Universidad de Sevilla, Spain

## Abstract

Rates of spontaneous mutation have been estimated under optimal growth conditions for a variety of DNA-based microbes, including viruses, bacteria, and eukaryotes. When expressed as genomic mutation rates, most of the values were in the vicinity of 0.003–0.004 with a range of less than two-fold. Because the genome sizes varied by roughly 10^4^-fold, the mutation rates per average base pair varied inversely by a similar factor. Even though the commonality of the observed genomic rates remains unexplained, it implies that mutation rates in unstressed microbes reach values that can be finely tuned by evolution. An insight originating in the 1920s and maturing in the 1960s proposed that the genomic mutation rate would reflect a balance between the deleterious effect of the average mutation and the cost of further reducing the mutation rate. If this view is correct, then increasing the deleterious impact of the average mutation should be countered by reducing the genomic mutation rate. It is a common observation that many neutral or nearly neutral mutations become strongly deleterious at higher temperatures, in which case they are called temperature-sensitive mutations. Recently, the kinds and rates of spontaneous mutations were described for two microbial thermophiles, a bacterium and an archaeon. Using an updated method to extrapolate from mutation-reporter genes to whole genomes reveals that the rate of base substitutions is substantially lower in these two thermophiles than in mesophiles. This result provides the first experimental support for the concept of an evolved balance between the total genomic impact of mutations and the cost of further reducing the basal mutation rate.

## Author Summary

Spontaneous mutations are key drivers of evolution and disease. In microbes, most mutations are deleterious, some are neutral (without significant impact), and a few are advantageous. Because deleterious mutations reduce fitness, there should be constant selection for antimutator mutations that reduce rates of spontaneous mutation. However, such reductions are necessarily achieved at some cost. Therefore, a mutation rate should converge evolutionarily on a value that reflects this trade-off. For DNA microbes, the observed genomic mutation rate is remarkably (and mysteriously) invariant, in the neighborhood of 0.003–0.004, with a range of less than two-fold despite huge variation per average base pair in organisms with a wide diversity of life histories. Would an environmental condition that increased the average deleterious impact of a mutation be balanced by additional investments in antimutator mutations? It is widely observed that many mutations with mild impacts become strongly deleterious at higher temperatures, so mutation rates were measured in two thermophiles, a bacterium and an archaeon. Remarkably, both displayed average mutation rates reduced by about five-fold from the characteristic mesophilic value, most of the decrease reflecting a 10-fold reduction in the rate of base substitutions.

## Introduction

It has become increasingly clear that the basal rate of spontaneous mutation per genome per replication is remarkably invariant in DNA microbes: using a classical correction factor for estimating the ratio of all base-pair substitutions (BPSs) to detected base-pair substitutions, genomic mutation rates (mutations per genome per replication) vary by less than twofold while genome sizes vary by ≈6,000-fold (Table 1). Thus, when mutation rates are expressed per average base pair, they also vary by a similarly large factor. Therefore, basal mutation rates characteristic of unstressed microbial populations can evolve to finely tuned values. The theory of mutation rates has its roots in Haldane's 1927 formulation of the impact of selection and mutation on fitness [1], followed by Sturtevant's 1937 conjecture that the deleterious character of most mutations would generate selective pressures that should lower mutation rates indefinitely [2]. In 1967, Kimura offered the hypothesis that there would be a “physiological cost” to each reduction in rate, leading to an equilibrium value when that cost outweighs the gain in fitness [3]. The surprise has been that the observed genomic rates are so narrowly distributed among DNA microbes despite a wide variety of life histories and genome sizes. An even deeper mystery, not to be addressed here, is why the particular microbial genomic rate of about 0.003–0.004 has been adopted by microbes of such diverse life histories and genome sizes.

**Table 1:** Microbial genomic mutation rates estimated using historical methods.

Organism	*G*	μ*_b_*	μ*_g_*
Phage M13	6.4×10^3^	7.5×10^−7^	0.0048
Phage λ	4.9×10^4^	6.6×10^−8^	0.0032
Herpes simplex virus	1.5×10^5^	1.8×10^−8^	0.0028
Phage T2	1.6×10^5^	2.7×10^−8^	0.0043
Phage T4	1.7×10^5^	2.8×10^−8^	0.0047
*Escherichia coli*	4.6×10^6^	7.9×10^−10^	0.0037
*Saccharomyces cerevisiae*	1.2×10^7^	2.9×10^−10^	0.0037
*Schizosaccharomyces pombe*	1.3×10^7^	3.2×10^−10^	0.0044
*Neurospora crassa*	3.8×10^7^	6.6×10^−11^	0.0028
Range	5,900-fold	11,000-fold	1.7-fold

*G* = genome size in bases or base pairs. μ*_b_* = average mutation rate per base pair per replication. μ*_g_* = mutation rate per genome per replication  = *G*×μ*_b_*. See the Calculations in the Methods for details.

If the Kimura conjecture is correct, then increasing the average deleterious impact of a spontaneous mutation (and thus converting many neutral or nearly-neutral mutations to deleterious mutations) would lower the rate of mutation, at least on an evolutionary time scale. The concept of an equilibrium basal mutation rate is difficult to test in a laboratory context because any imposed resetting of the equilibrium would probably require numbers of generations large even by microbial standards, and is difficult to test convincingly because only one or a few habitats could be explored. However, it has recently proven possible to test the concept by examining a natural evolutionary experiment, life at high temperatures. Those who gather mutants for fun or profit have often observed that the most common class of mutations is to temperature sensitivity, indicating that many missense mutations are well tolerated at the standard growth temperature but become much more deleterious, often to the point of lethality, at a temperature only 5°C–10°C higher. This widespread anecdotal observation implies that macromolecular stability becomes increasingly dependent on structural integrity as temperatures rise, a reasonable conjecture in keeping with the considerable constraints observed in the proteins of thermophilic microbes (e.g., [4]). It is therefore likely that the average missense mutation harms thermophiles more than mesophiles (the *hypothesis of dangerous missense*). This simple prediction was supported by the observation that missense mutations accumulated to a lesser extent (compared to synonymous mutations) in thermophiles than in mesophiles during the course of molecular evolution (d_N_/d_S_ falling from 0.14 to 0.09), implying stronger purifying selection in thermophiles [5]. Here, direct measurements of the rate and character of spontaneous mutation are compared for mesophilic and thermophilic microbes.

## Results

### Two Extrapolation Problems

The first phase of determining genomic mutation rates involves measuring a mutation frequency, converting the frequency to a rate, and taking precautions to exclude or take into account the impact of perturbations such as differential growth rates of mutants versus wild type and delayed expression of the mutant phenotype. In addition to measuring rates, it is crucial to identify the kinds of mutations that arise in order to exclude biases due to massive mutational hotspots or to bizarre classes of mutations. The typical result is a rate for a mutation-reporter gene, which is then extrapolated to the whole genome provided that the spectrum of mutations is fairly ordinary. However, there is a substantial problem here: while most indels are detected, most BPSs fail to produce a phenotypic change detectable in the laboratory. One must therefore estimate their full frequencies. (An exception is the still rare case that mutation detection is achieved with phenotype-blind genomic DNA sequencing.) Two methods have been applied. Both make the reasonable assumption that almost all indels and chain-termination (CT) BPSs are detected with high efficiency in protein-coding sequences. (Although exceptions occur, they are infrequent and tend to occur at the extreme downstream end of a gene.) The first method was based in part on the average relative frequencies of CT and non-CT BPSs in a handful of spectra and provided a correction factor for base substitutions of 4.726 [6]. This method was used for almost all of the entries in Table 1; however, the range of values averaging to 4.726 was large, reducing reliability. The second method is based exclusively on CT mutations. It involves examining the reporter sequence for all possible BPSs capable of generating CTs and then dividing the observed CT mutation frequency by that reduced target size and multiplying by 3 (to account for the three BPSs that can arise at any site) to obtain an average mutation rate per base pair. The CT method also has drawbacks. First, it cannot report A·T→G·C mutations, but these generally arise at approximately average BPS rates, suggesting a minimal problem. Second, CT mutations are typically a minority of all mutations, so that many spectra sport only a few CTs, reducing sampling accuracy.

The other major barrier to accurate extrapolation from a mutation-reporter gene to the whole genome becomes manifest when sequencing reveals a major hotspot. Mutation rates at particular sites vary greatly, but most mutational spectra display a range of site-specific numbers of mutations ranging from 1 to hotspots with from several percent to even a quarter of the whole collection. The impact of a hotspot containing a quarter of all the mutations is modest, but some genes contain single hotspots bearing the large majority of mutations; the classic example is the *E. coli lacI* gene, where ∼72% of all mutations are indels arising at a stretch of 13 BPSs consisting of 3.25 repeats of a tetramer [7]. However, such massive indel hotspots are infrequent among genes, and it is reasonable to post occasional genomic rates both including and removing them.

### Thermophiles Versus Mesophiles

All informative microbial mutation rates obtained before 2000 were for mesophilic species, but rates and spectra are now available for two genes in each of two very different thermophiles, the crenarchaeon *Sulfolobus acidocaldarius* [6] and the bacterium *Thermus thermophilus* [8], both growing at close to 75°C. In the first study, with *S. acidocaldarius*, BPSs were a smaller fraction of the spectrum than in mesophiles, and this observation prompted the hypothesis of dangerous missense. Note, however, that if greater fractions of missense mutations are phenotypically detectable in thermophiles than in mesophiles, then the historical method of correcting for undetected BPSs becomes inappropriate when based on mesophiles. It is therefore advisable to resort exclusively to the CT method for estimating total BPS rates, which is the central result for this report.

Table 2 lists genomic mutation rates estimated using the CT method (or its *lacZ*α equivalent), sometimes based on the same sources as for Table 1 but excluding some reports whose sequencing information was inadequate for the CT method. The nine entries at the top are for mesophiles and reveal no significant departures from the values in Table 1, providing empirical confidence in the robustness of the CT method. The two entries at the bottom are for thermophiles, whose numbers of CTs are small. (The data for the two mutation-reporter genes are combined in each organism because of the small number of CTs.) The thermophile BPS rates are substantially lower, by about 10-fold, than their mesophile counterparts. When major indel hotspots are included, indel rates are less than twofold lower in thermophiles, while total genomic rates are about fivefold lower. (When the indel hotspots are removed from the analysis, the indel rate decrease is three-fold and the total genomic rate decrease is seven-fold.) Although these ratios are somewhat uncertain because of the small numbers of CTs for five of the seven mesophiles and both thermophiles, the mean difference is large enough to support the inference that BPS rates are lower in thermophiles. The mesophile and thermophile values were compared using randomization t-tests [9], a nonparametric test that requires no assumptions about normality or equal variances of the mutation rates. The resulting one-sided *p* values are 0.018 for both the total mutation rate and its BPS component, and 0.27 for the indel values that include the hotspots.

**Table 2:** Microbial genomic mutation rates calculated using the CT method.

Organism	Mutation reporter^a^	μ*_g_*(*I*) (−HS)^b^	μ*_g_*(*B*) (CTs)^c^	μ*_g_* (−HS)^b^
Phage M13	*lacZα*	0.00103	0.0038 (245)	0.0048
Phage λ	*cII*	0.00041	0.0022 (8)	0.0026
Phage T4	*rI*	0.00079	0.0030 (6)	0.0038
Herpes simplex virus	*tk*	0.00083	0.0035 (5)	0.0043
*Escherichia coli*	*lacI*	0.00230 (0.00042)	0.0025 (24)	0.0048 (0.0030)
*Saccharomyces cerevisiae*	*URA3* (4)	0.00016	0.0029 (108)	0.0030
	*CAN1* (3)	0.00056	0.0058 (76)	0.0063
*Schizosaccharomyces pombe*	*ura4*	0.00031	0.0019 (5)	0.0022
	*ura5*	0.00050	0.0034 (4)	0.0039
**Mesophile mean**		**0.00077** (0.00056)	**0.0032**	**0.0040** (0.0038)
Mesophile range		15-fold (6.7-fold)	3.0-fold	2.9-fold (2.9-fold)
				
*Sulfolobus acidocaldarius*	*pyrEF*	0.00053 (0.00026)	0.00011 (1)	0.00065 (0.00037)
*Thermus thermophilus*	*pyrEF*	0.00038 0.00013	0.00054 (2)	0.00093 (0.00067)
**Thermophile mean**		**0.00046** (0.00019)	**0.00033**	**0.00079** (0.00052)
Thermophile range		1.4-fold (2.0-fold)	4.8-fold	1.4-fold (1.8-fold)
				
**Mean (mesophile/thermophile)**		**1.7** (2.9)	**9.8**	**5.1** (7.2)

μ*_g_*(*I*) = genomic indel rate. μ*_g_*(*B*) = genomic base-pair substitution rate. μ*_g_* = μ*_g_*(*I*)+μ*_g_*(*B*).

a (Number of values averaged).

b (Value excluding a large frameshift hotspot).

c (Number of chain-terminating mutations or equivalent).

## Discussion

### The Central Result

Genomic mutation rates have long been suspected to evolve as a balance between the deleterious impact of the average mutation and the cost of further reducing the mutation rate. A test of this conjecture on the evolutionary scale could consist of estimating mutation rates in organisms whose environment increases the impact of the average mutation. Because many base substitutions do greater harm at higher temperatures, thermophiles were suitable candidate organisms. For both a bacterium and an archaeon, the thermophiles display sharply reduced rates of base pair substitutions compared to the typical mesophile.

The lower mutation rates in thermophiles are likely to reflect their higher optimal growth temperatures. There is no obvious hint of a particular aspect of life history other than temperature that sets the two thermophiles apart from the mesophiles. The %(G+C) values for the ten organisms in Tables 1 and/or 2, listed monotonically with the two thermophile values in bold, are 35–36–**37**–38–41–50–50–51–68–**69**, providing no hint of a role for this variable, as also noted in the earlier molecular-evolution study [5]. Thus, the Kimura conjecture, that the equilibrium mutation rate reflects a balance between the impact of the average mutation compared to the cost of keeping mutations in check, is supported in a natural experiment.

The hypothesis of dangerous missense predicts that BPS rates will be reduced in thermophiles but does not speak directly to indel rates. However, indel rates are also reduced, although less strongly than are BPS rates and with a *p* value of 0.27 for these data. One candidate explanation for this difference is that the reduction in BPS rates is achieved by the accumulation of modifiers selected to target BPS mutagenesis but at most incidentally targeting indel mutagenesis. Because single-base additions and deletions tend to be the large majority of indels in mesophiles (35 single-base indels/38 total indels in phage λ, 20/23 in phage T4, 45/45 in Herpes simplex virus, 604/641 in *E. coli*, 88/97 in *S. cerevisiae*, and 24/32 in *S. pombe*) and are similarly frequent in thermophiles (84/95 in *S. acidocaldarius* and 46/54 in *T. thermophilus*), these small indels must be the main targets of antimutagenic modifiers acting on indels generally. Both single-base indels and BPSs result from errors of insertion followed by failures of proofreading and DNA mismatch repair in well studied model organisms such as *E. coli* and *S. cerevisiae*, but little is known about the sources of spontaneous mutations in *S. acidocaldarius* and *T. thermophilus*.

### New Fishing Holes

Are there likely to be other outliers with informative deviations from the mutational pattern that is consistently displayed among the mesophilic microbes examined to date with respect to either the mutation rate or the BPS:indel ratio?

Mutations to cold sensitivity are rarely reported and are anecdotally described as difficult to discover. If they are indeed rare, perhaps fewer missense mutations produce mutant phenotypes in psychrophiles than in mesophiles. One evolutionary consequence might then be a relaxation to a higher spontaneous rate of BPS mutation, perhaps with little effect on the rate of indel mutation.

Because of incomplete buffering against the impacts of their environments, halophiles and acidophiles experience relative high internal concentrations of Na^+^ and H^+^, respectively, compared to other microbes. These ionic environments might be unusually stressful to mutants carrying missense mutations, resulting in adjustments to their mutational patterns in the same direction as seen for thermophiles. Although without significance because of sampling constraints, Table 2 attributes a five-fold lower BPS mutation rate to the acidophile *S. acidocaldarius* than to the non-acidophile *T. thermophilus*. Unfortunately, an attempt to characterize mutation in the halophilic archaeon *Haloferax volcanii* failed, probably because this mesophile is highly polyploid [10].

The lactic acid bacterium *Oenococcus oeni*, used in wine making to convert malic acid to lactic acid, lacks the usual bacterial DNA mismatch repair (MMR) system and has a high mutation rate as judged by mutations conferring resistance to rifampin and erythromycin, as does *Oenococcus kitaharae* [11]. These results suggest a powerful genus-wide mutator condition, which would normally be highly deleterious. The question then arises whether the lack of MMR is so strongly adaptive in these species as to outweigh the sharply decreased fitness of the mutator condition, or whether the species have been unable to re-acquire the MMR genes by horizontal transfer.

Whereas the above two species lack MMR function and display mutator phenotypes, the crenarchaeons as a whole, including *S. acidocaldarius*, lack all known bacterial MMR genes, but *S. acidocaldarius*, at least, displays an antimutator phenotype compared with mesophiles. How can this be? In *Escherichia coli*, the mutation rate per average base pair ≈8×10^−10^ (Tables 1 and 2). Based on the strengths of mutator mutations, replication infidelity can be estimated as the product of three components during DNA replication: insertion errors ≈0.9×10^−5^, proofreading failures ≈1.7×10^−2^, and MMR failures ≈5×10^−3^ [12],[13]. In bacteriophage T4, which does not employ a general MMR system, the mutation rate per average base pair ≈2×10^−8^ (Tables 1 and 2). Based on the strengths of mutator mutations, replication fidelity can be estimated as the product of two components during DNA replication: insertion errors ≈1×10^−5^ and proofreading failures ≈2×10^−3^ [13]. Thus, T4 makes up for the lack of MMR by a proofreading potency about an order of magnitude greater than that operating in *E. coli*. The mutation rate per base pair for *S. acidocaldarius* ≈3×10^−10^, which might be achieved by a product of factors applied to the T4 insertion and proofreading accuracies that together produce a 70-fold improvement. Alternatively, *S. acidocaldarius* may possess an MMR system so distinct from the standard *mutHLS* model as to have escaped recognition by genomic scans. Note also that both thermophiles have genomes about twofold smaller than the *E. coli* genome.

## Methods

### General Procedures

We begin in possession of values for the following:

*G* = the genome size in bases or base pairs.

*T* = the number of bases or base pairs in the target (the mutation-reporter sequence).

μ_T_ = the measured mutation rate at *T*, corrected where necessary for mutants expressing the characteristic phenotype but revealed by sequencing to lack mutations in the reporter gene, but not corrected for mutants with two or more mutations (which are infrequent and sometimes absent). In many cases, μ*_T_* = *f*/ln(μ*_T_N*) where *f* = the measured mutation frequency for the given target, *N* = the final population size, and the median μ*_T_* over several cultures is used [14], a method that is robust compared to the classical fluctuation test provided the average number of mutational events per culture is ≥30 [15].

*M* = number of sequenced mutants = *B*+*I*, where *B* = number of BPS mutants and *I* = number of indel mutants, the latter also including complex mutants (a minority, if present at all) regardless of their components.

For the “historical” method, we correct for undetected BPSs by multiplying the number of detected BPSs by 4.726 [6]. Then the average mutation rate per base or base pair μ*_b_* = [μ*_T_* corrected upwards by (*I*+4.726*B*)/*M*]/*T* = (*I*+4.726*B*)( μ*_T_*/*MT*). The genomic mutation rate μ*_g_* = *G*μ*_b_*.

For the “CT” method, the indel genomic mutation rate μ*_g_*(*I*) is calculated as above ignoring the BPS component, *B* becomes *B_CT_* = number of mutations to a chain-terminating codon (TAG, TGA, or TAA), and *P* = number of possible mutational pathways to a CT mutation within *T* (there being three mutational BPS pathways per base or base pair). Then the BPS genomic mutation rate μ*_g_*(*B*) = μ*_T_* (3*B_CT_*/*MP*)*G*. The total genomic rate μ*_g_* = μ*_g_*(*I*)+μ*_g_*(*B*).

### Calculations

#### Phage M13

*G* = 6407. This system is unique among popular mutation reporters. It consists of an *E. coli lacZ*α transgene embedded in the single-stranded DNA of the M13 genome and carrying both an upstream regulatory region and the beginning of the *lacZ* gene. Because thousands of mutants have been sequenced, it has become apparent which mutations are detectable when present singly and which are not [14],[16]. The target sizes for base substitutions (*T_B_* = 245) and for single-base indels (*T*_±1_ = 177) are thus well defined, and we further assume that the infrequent larger indels are fully detectable (*T_L_* = 239). The measured mutation frequency *f* was 5.86×10^−4^ [17], *M* = 117, *B* = 67, *I*_±1_ = 11 and *I_L_* = 39. Assuming that virtually all replication occurs by a rolling circle mechanism, the mutation rate is calculated as for RNA viruses, μ = *f*/2*c* where *c* is the number of consecutive cycles of infection [18]. The following protocol was used to grow the stock ([17] and T. A. Kunkel, personal communication). The contents of one plaque (≥10^13^ pfu) were added to l L of medium containing *E. coli* cells diluted from an overnight culture to about 10^7^ cells/ml, so that the multiplicity of infection was about 10^3^. The input of infected cells from the plaque was ≤10^8^, so that the input concentration of infected cells  = 10^8^/10^3^/ml = 10^5^/ml, that is, no more than 10^5^/10^7^ = 0.01 of all cells. *c*≈2.5 in the plaque +1 in the liquid culture  = 3.5. Then μ*_b_* = (*f*/2*c*)Σ(proportion of mutations of type *i* = *N_i_*/117)(1/*T_i_*) = (5.86×10^−4^/7)[(3×67/117)(1/245)+(11/117)(1/177)+(39/117)(1/239)] = 7.48×10^−7^. μ*_g_*(*B*) = (*f*/2*c*)(3×proportion of BPSs)(*G*/*T_B_*) = (5.86×10^−4^/7)(3×67/117)(6407/245) = 0.00376, μ*_g_*(*I+L*) = (*f*/2*c*)[(proportion of ±1 indels)(1/*T*_±1_)+(proportion of larger indels)(1/*T_L_*)](*G*) = (5.86×10^−4^/7)[(11/117)(1/177)+(39/117)(1/239)](6407) = 0.00103, and μ*_g_* = μ*_g_*(*B*)+μ*_g_*(*I+L*) = 0.00479.

#### Phage λ

*G* = 4.850×10^4^. For the *cII* gene, *T* = 294, *f* = 5.36×10^−5^, and, for 93 mutants, *B* = 55 (*B_CT_* = 8 and *P* = 35) and *I* = 38 ([19] and J. Wagner, personal communication). Then μ*_T_* = *f*/ln(μ*_T_ N*) = 6.10×10^−6^. Using the historical method, μ*_b_* = (6.10×10^−6^)(38+55×4.726)(1/93)(1/294) = 6.64×10^−8^ and μ*_g_* = 0.00304. μ*_g_*(*I*) = (6.10×10^−6^)(38/93)(4.85×10^4^/294) = 0.00041. Using the CT method, μ*_g_*(*B*) = (6.10×10^−6^)(8/93)(3×4.85×10^4^/35) = 0.00218 and μ*_g_* = μ*_g_*(*I*)+μ*_g_*(*B*) = 0.00259.

#### Herpes simplex virus type 1

*G* = 1.523×10^5^. For the *tk* gene, *T* = 1131, *f* = 6×10^−5^, *N* = (0.3–20)×10^8^ and, for 67 mutants, *B* = 22 (*B_CT_* = 5 and *P* = 90) and *I* = 45 [20]. Replication proceeds by a mixture of exponential and linear replication, for which we use μ*_T_* = *f*/ln(μ*_T_N*) and μ*_T_* = *f*/2*c* (with *c* = 2), respectively. The corresponding values are μ*_T_* = 8.40×10^−6^ and 10×10^−6^, giving a mean of 9.20×10^−6^. Using the historical method, μ*_b_* = (9.20×10^−6^)(45+22×4.726)(1/67)(1/1131) = 1.81×10^−8^ and μ*_g_* = 0.00275. μ*_g_*(*I*) = (9.20×10^−6^)(45/67)(1.52×10^5^/1131) = 0.00083. Using the CT method, μ*_g_*(*B*) = (9.20×10^−6^)(5/67)(3×1.52×10^5^/90) = 0.00348 and μ*_g_* = μ*_g_*(*I*)+μ*_g_*(*B*) = 0.00432.

#### Phage T2

See [15].

#### Phage T4

*G* = 1.689×10^5^. For the *rI* gene, *T* = 294, μ*_T_* = 2.82×10^−6^ and, for 66 mutants, *B* = 34 (*B_CT_* = 6 and *P* = 43) and *I* = 32 [21]. Using the historical method, μ*_b_* = (2.82×10^−6^)(32+34×4.726)(1/66)(1/294) = 2.80×10^−8^ and μ*_g_* = 0.00473. μ*_g_*(*I*) = (2.82×10^−6^)(32/66)(1.689×10^5^/294) = 0.00079. Using the CT method, μ*_g_*(*B*) = (2.82×10^−6^)(6/66)(3×1.69×10^5^/43) = 0.00302 and μ*_g_* = μ*_g_*(*I*)+μ*_g_*(*B*) = 0.00381.

#### *Thermus thermophilus*

*G* = 2.127×10^6^. For the *pyrEF* genes, *T* = 1326, μ*_T_* = 3.21×10^−7^ and, for 73 mutants, *B* = 19 (*B_CT_* = 2 and *P* = 103) and *I* = 54 (or 18 without the hotspot) [8]. The historical method is inappropriate for thermophiles (see text). μ*_g_*(*I*) = (3.21×10^−7^)(54/73)(2.127×10^6^/1326) = 0.000381. Using the CT method, μ*_g_*(*B*) = (3.21×10^−7^)(2/73)(3×2.127×10^6^/103) = 0.000545. μ*_g_* = μ*_g_*(*I*)+μ*_g_*(*B*) = 0.000926 (or 0.000672 without the indel hotspot).

#### *Sulfolobus acidocaldarius*

*G* = 2.226×10^6^. For the *pyrEF* genes, *T* = 1240, μ*_T_* = 3.37×10^−7^ and, for 108 mutants, *B* = 13 (*B_CT_* = 1 and *P* = 184) and *I* = 95 (or 46 without the hotspot) [6]. The historical method is inappropriate for thermophiles (see text). μ*_g_*(*I*) = (3.37×10^−7^)(95/108)(2.226×10^6^/1240) = 0.000532. Using the CT method, μ*_g_*(*B*) = (3.37×10^−7^)(1/108)(3×2.226×10^6^/184) = 0.000113. μ*_g_* = μ*_g_*(*I*)+μ*_g_*(*B*) = 0.000645 (or 0.000371 without the indel hotspot).

#### *Escherichia coli*

*G* = 4.639×10^6^. For the *lacI* gene, μ*_T_* = 6.043×10^−7^ (excluding 10 *IS* insertions) [15]. *T* = 1083 and, for 721 mutants, *B* = 80 (*B_CT_* = 24 and *P* = 110) and *I* = 641 (or 116 without the indel hotspot) [7]. Using the historical method, μ*_b_* = (6.043×10^−7^)(641+80×4.726)(1/721)(1/1083) = 7.89×10^−10^ and μ*_g_* = 0.00366. μ*_g_*(*I*) = (6.043×10^−7^)(641/721)(4.639×10^6^/1083) = 0.00230. Using the CT method, μ*_g_*(*B*) = (6.043×10^−7^)(24/721)(3×4.639×10^6^/110) = 0.00255 and μ*_g_* = μ*_g_*(*I*)+μ*_g_*(*B*) = 0.00485 (or 0.002961 without the indel hotspot).

#### *Saccharomyces cerevisiae*

*G* = 1.246×10^7^ and calculations are as above.

For the *URA3* gene, *T* = 804, *P* = 123, and four sets of values are available. For the first [22], μ*_T_* = 2.77×10^−8^ and, for 106 mutants, *B* = 89 (*B_CT_* = 39) and *I* = 17; for the historical method, μ*_b_* = 1.42×10^−10^ and μ*_g_* = 0.00177; μ*_g_*(*I*) = 0.00007; for the CT method, μ*_g_*(*B*) = 0.00310; and μ*_g_* = 0.00317. For the second [23], μ*_T_* = 6.25×10^−8^ and, for 20 mutants, *B* = 15 (*B_CT_* = 4) and *I* = 5; for the historical method, μ*_b_* = 2.95×10^−10^ and μ*_g_* = 0.00368; μ*_g_*(*I*) = 0.00024; for the CT method, μ*_g_*(*B*) = 0.00380; and μ*_g_* = 0.00404. For the third [23], μ*_T_* = 3.50×10^−8^ and, for 106 mutants, *B* = 89 (*B_CT_* = 39) and *I* = 17; for the historical method, μ*_b_* = 1.56×10^−10^ and μ*_g_* = 0.00195; μ*_g_*(*I*) = 0.00017; for the CT method, μ*_g_*(*B*) = 0.00022; and μ*_g_* = 0.00038. For the fourth [24], μ*_T_* = 4.75×10^−8^ and, for 106 mutants, *B* = 89 (*B_CT_* = 39) and *I* = 17; for the historical method, μ*_b_* = 2.37×10^−10^ and μ*_g_* = 0.00295; μ*_g_*(*I*) = 0.00014; for the CT method, μ*_g_*(*B*) = 0.00446; and μ*_g_* = 0.00460. The respective averages are, for the historical method, μ*_b_* = 2.08×10^−10^ and μ*_g_* = 0.00259; μ*_g_*(*I*) = 0.00015; and, for the CT method, μ*_g_*(*B*) = 0.00289 and μ*_g_* = 0.00305.

For the *CAN1* gene, *T* = 1773, *P* = 226, and three sets of values are available. For the first [25], μ*_T_* = 2.77×10^−7^ and, for 20 mutants, *B* = 11 (*B_CT_* = 1) and *I* = 9; for the historical method, μ*_b_* = 5.18×10^−10^ and μ*_g_* = 0.00645; μ*_g_*(*I*) = 0.00095; for the CT method, μ*_g_*(*B*) = 0.00249; and μ*_g_* = 0.00344. For the second [26], μ*_T_* = 3.01×10^−7^ and, for 23 mutants, *B* = 17 (*B_CT_* = 5) and *I* = 6; for the historical method, μ*_b_* = 4.13×10^−10^ and μ*_g_* = 0.00514; μ*_g_*(*I*) = 0.00036; for the CT method, μ*_g_*(*B*) = 0.00701; and μ*_g_* = 0.00737. For the third [24], μ*_T_* = 1.52×10^−7^ and, for 227 mutants, *B* = 150 (*B_CT_* = 70) and *I* = 77 (including 13 complex mutations); for the historical method, μ*_b_* = 2.97×10^−10^ and μ*_g_* = 0.00370; μ*_g_*(*I*) = 0.00036; for the CT method, μ*_g_*(*B*) = 0.00775; and μ*_g_* = 0.00811. The respective averages are, for the historical method, μ*_b_* = 4.09×10^−10^ and μ*_g_* = 0.00510; μ*_g_*(*I*) = 0.00056; and, for the CT method, μ*_g_*(*B*) = 0.00575 and μ*_g_* = 0.00631.

The averages of the four *URA3* plus three *CAN1* values (sum÷7) are: for the historical method, μ*_b_* = 2.94×10^−10^ and μ*_g_* = 0.00366; μ*_g_*(*I*) = 0.00033; and, for the CT method, μ*_g_*(*B*) = 0.00412 and μ*_g_* = 0.00444.

#### *Schizosaccharomyces pombe*

*G* = 1.252×10^7^, values are from [27] and S. Davey (personal communication) and calculations are as above. For the *ura4* gene, *T* = 795, μ*_T_* = 4.56×10^−8^ and, for 39 mutants, *B* = 22 (*B_CT_* = 5, *P* = 116) and *I* = 17; for the historical method, μ*_b_* = 1.78×10^−10^ and μ*_g_* = 0.00223; μ*_g_*(*I*) = 0.00031; for the CT method, μ*_g_*(*B*) = 0.00189; and μ*_g_* = 0.00221. For the *ura5* gene, *T* = 648, μ*_T_* = 8.44×10^−8^ and, for 49 mutants, *B* = 34 (*B_CT_* = 5, *P* = 96) and *I* = 15; for the historical method, μ*_b_* = 4.67×10^−10^ and μ*_g_* = 0.00585; μ*_g_*(*I*) = 0.00050; for the CT method, μ*_g_*(*B*) = 0.00337; and μ*_g_* = 0.00387. The average values for the two genes are: for the historical method, μ*_b_* = 3.23×10^−10^ and μ*_g_* = 0.00404; μ*_g_*(*I*) = 0.00041; for the CT method, μ*_g_*(*B*) = 0.00263; and μ*_g_* = 0.00304.

#### **Neurospora crassa:**

*G* = 3.804×10^7^. Using the old mutation data (see [15]), for *ad-3AB*, μ*_b_* = 4.11×10^−11^ and μ*_g_* = 0.00172. For *mtr*, μ*_b_* = 9.15×10^−11^ and μ*_g_* = 0.00383. The average values are μ*_b_* = 6.63×10^−11^ and μ*_g_* = 0.00278.
